# Spatial-temporal differentiation of urban eco-efficiency and its driving factors: A comparison of five major urban agglomerations in China

**DOI:** 10.1371/journal.pone.0300419

**Published:** 2024-03-21

**Authors:** Xiang Liu, Xinyuan Zhang, Man Yuan, Jia Liu, Guolin Zhou

**Affiliations:** 1 School of Business Administration, Guangdong University of Finance, Guangzhou, China; 2 School of Information and Safety Engineering, Zhongnan University of Economics and Law, Wuhan, China; 3 School of Journalism and Culture Communication, Zhongnan University of Economics and Law, Wuhan, China; Shenzhen University, CHINA

## Abstract

This paper utilizes an improved undesirable output DEA model to measure the eco-efficiency of cities in five major urban agglomerations in China during 2006–2020. It employs the Theil Index and Geodetector to investigate the spatial-temporal distribution differentiation characteristics and driving factors of urban eco-efficiency. The main findings are as follows. Firstly, the eco-efficiency of all urban agglomerations showed a fluctuating upward trend, but the eco-efficiency performance of different urban agglomerations in China shows a stratification characteristic. Specifically, the Pearl River Delta urban agglomeration consistently ranks first in China, while the mean values of the Yangtze River Delta urban agglomeration, Beijing-Tianjin-Hebei urban agglomeration, and Chengdu-Chongqing urban agglomeration are lower than the national average. Secondly, the overall differences in the urban eco-efficiency of all sample cities show a consistently fluctuating downward trend. The factor that affects the level differences of eco-efficiency in different cities is the intra-regional differences. Last but not least, the top three factors affecting the spatial distribution difference of urban eco-efficiency in the whole sample are environmental pollution control investments, innovation level, and environmental infrastructure investments. In the end, this paper proposes that reducing the intra-regional differences is the primary task to achieve the coordinated improvement of urban eco-efficiency in urban agglomerations, and then puts forward some policy suggestions to improve eco-efficiency for the five major urban agglomerations.

## 1. Introduction

As the highest spatial organizational form for economic activities, urban agglomerations are replacing the traditional provincial economic development pattern in China. The National New Urbanization Plan (2014–2020) was implemented, inducing the urban agglomeration to play a dominating role in protecting the ecological environment and achieving sustainable development in the long run. In the environmental governance and green development of urban agglomerations, environmental pollution control actions are mainly implemented separately based on local interests [1]. Therefore, the National Plan for New Urbanization of China (2021–2035) proposes to promote coordinated and interconnected environmental governance among cities in different regions, using urban agglomerations as the main platform. The plan underscores the imperative role of urban agglomerations in facilitating regional coordinated development. Specifically, it highlights the significance of 19 national-level urban agglomerations. Among them, five mature urban agglomerations, namely Beijing-Tianjin-Hebei, Yangtze River Delta, Pearl River Delta, Chengdu-Chongqing, and the Central Yangtze River Region belong to the “optimization and upgrading scope” and have maintained a leading position in the development of urban clusters in China. Remarkably, in 2020, these five major urban agglomerations accounted for a meager 11% of China’s total land area but concentrated 42% of the nation’s population and generated an impressive GDP of 54.8 trillion RMB, equivalent to 54% of the national GDP. They have emerged as the primary driving force propelling China’s economic growth, effectively harnessing regional agglomeration and scale effects. In particular, China has proposed building a hierarchical urban agglomeration system with five national-level large urban agglomerations, nine regional-level medium-sized urban agglomerations, and six sub-regional-level small-sized urban agglomerations. The five large urban agglomerations are the current foci of China’s New Urbanization strategy. They include the Yangtze Delta urban agglomeration, Pearl River Delta urban agglomeration, Jing-Jin-Ji urban agglomeration, Yangtze River Middle-Reach urban agglomeration, and Cheng-Yu urban agglomeration.

The development of urban agglomerations in China exhibits the following characteristics: rapid economic growth, high agglomeration efficiency, extensive functional radiations, and strong regional interconnections [2]. However, the unevenness of their development is gradually becoming evident. From a national perspective, the discrepancies in development among various urban agglomerations in China are particularly noticeable because of variations in resource endowment, geographic position, degree of openness, innovation potential, and talent attractiveness [2].

Measuring the eco-efficiency of different urban agglomerations in China is the logical starting point for exploring the green development path of urban agglomerations. Comparing the eco-efficiency difference between and within urban agglomerations is the key to achieving the balanced development of urban agglomerations under resource and environment constraints, which directly affects the coordinated regional economic development of urban agglomerations in China.

Therefore, assessing urban eco-efficiency from the perspective of urban agglomerations, and exploring the spatial differences and influencing factors of urban eco-efficiency within urban agglomerations have gradually become the interests of many scholars. Consequently, this paper intends to conduct a longitudinal and horizontal comparative analysis of urban eco-efficiency levels and the characteristics of the spatial-temporal distribution differences and their driving factors to provide policy recommendations for improving eco-efficiency in urban agglomerations. The selected five major agglomerations are the Yangtze River Delta, Pearl River Delta, Beijing-Tianjin-Hebei, Chengdu-Chongqing, and the middle reaches of the Yangtze River urban agglomeration. National New-type Urbanization Plan (2014–2020) positions these five urban agglomerations as national-level urban agglomerations because they were established earliest, possess the strongest economic power and influence, are the most mature in development, and are the most representative among urban agglomerations in China. They will play a more important role in the new pattern of urban space in China and are expected to explore new forms for China’s sustainable economic development.

## 2. Literature review

In the context of green development and high-quality development, urban development must focus on obtaining as many economic benefits as possible with as little environmental resource input as possible to achieve the unity of economic efficiency and environmental benefits. Therefore, scholars have proposed ecological efficiency indicators to measure and evaluate the green development level of cities [3, 4]. The notion of “eco-efficiency” refers to the empirical relationship between economic activities and environmental costs [5]. Since eco-efficiency essentially reflects the effectiveness of input and output, the study of urban eco-efficiency has become a research hotspot [6–8].

As for the measurement of eco-efficiency, the early evaluation methods of eco-efficiency can be mainly divided into two main categories: single indicator and indicator system. The former includes the ratio of GDP to the ecological environmental effect [9] and the ratio of the product value and product life to the environmental impact [10]. and the latter mainly includes the comprehensive indicator system [11, 12]. However, although the single indicator is simple to calculate, it contains limited information and cannot effectively distinguish the impact of different environments on ecological efficiency [7, 13]. The construction of the comprehensive indicator system, on the other hand, is based on integrating multiple indicators for comprehensive assessment, which has the advantages of a wide range of involvement, comprehensive consideration, and comparability in time and space. However, there is inevitably human subjectivity in selecting indicators, and the changes in indicators significantly impact the evaluation results. Moreover, both the single indicator and comprehensive indicator system reflect the economic performance from the perspective of the final results while ignoring the efficiency indicators in the process of economic development. Compared with the outcome indicators, the efficiency indicators can better reflect the ability to use resources in the process of economic development, i.e., the ability to achieve maximum output under the conditions of given various input factors or to minimize input under the given output level [1, 14]. The efficiency indicators are better matched to the goal of conducting high-quality economic development.

Due to its advantages in efficiency evaluation, the DEA model has become a mainstream method for eco-efficiency evaluation in recent years [14–19]. The traditional BCC-DEA model based on the production theory is considered from the perspective of a homogeneous increase in output or a homogeneous decrease in input. There are a series of problems, such as radial deviation, the large difference between measurement and actual production process, ignoring the spatial heterogeneity of DMU (Decision Making Unit), and difficulty in inter-period comparison. Moreover, the constraint with an efficiency value of 1 cannot distinguish the relative merits and demerits of these DMUs’ efficiency values [1]. To solve the problem of the BCC-DEA model, Banker, and Gifford separated the effective DMU from the reference efficiency frontier and constructed the super-efficiency DEA model, which has been widely applied to the measurement of eco-efficiency. In addition, considering the assessment of eco-efficiency, the environmental output must be taken into account. Many scholars have conducted detailed discussions on the problems of undesirable output variables, such as SO2 and CO2, and proposed the SBM-DEA (Slack-based Measure DEA) model containing the undesirable output [20–22]. The SBM-DEA model can identify the difference between undesirable output and desirable output variables, and define the maximum desirable output and minimum undesirable output under the fixed input level as the optimal efficiency. It also considers the problem of slack in undesirable and desirable output, while the obtained slack variables have a clear economic significance. Zhou et al. (2006, 2008) [23, 24] reviewed the application of the SBM-DEA model based on the undesirable output in environmental resources.

In the context of coordinated development of different regions, the importance of using urban agglomeration as a platform to promote integrated economic development and joint environmental protection has become increasingly prominent. Assessing urban eco-efficiency from the perspective of urban agglomeration and exploring the spatial differences and influencing factors of urban eco-efficiency within urban agglomerations have gradually become the focus of academics. Researchers have investigated the eco-efficiency and its evolution trend in Beijing-Tianjin-Hebe urban agglomeration [25], Yangtze River Economic Belt urban agglomeration [18, 26, 27], Pearl River Delta urban agglomeration [28], and Chengdu-Chongqing urban agglomeration [29]. Research on factors affecting eco-efficiency is mainly focused on the level of economic development, technological progress, industrial structure, opening degree, and environmental regulations [12, 15, 30, 31].

The theoretical and empirical research on urban eco-efficiency has laid a foundation for achieving high-quality urban development. However, there are still some limitations as follows. (1) Most of the current literature focuses on the inter-provincial and urban levels. The studies on urban agglomerations only focus on the analysis of a single urban agglomeration but not the comparison between different urban agglomerations, which makes it difficult to reflect the development differences among the urban agglomerations. (2) Current studies have not sufficiently considered the undesirable output based on environmental factors, either failing to consider the slackness of input and output or over-considering the slackness of variables, which leads to the negative impact of non-effective decision-making units on the improvement of eco-efficiency. (3) Current research has hardly considered the driving factors affecting the eco-efficiency levels of different urban agglomerations, which are closely related to the urban spatial correlation within the urban agglomerations. Therefore, the eco-efficiency improvement strategies under the high-quality development goals proposed by the existing studies need further improvement.

Responding to these deficiencies, this paper constructs a DEA model considering the undesirable output. Five major urban agglomerations in China are selected, including the Yangtze River Delta, Pearl River Delta, Beijing-Tianjin-Hebei, Chengdu-Chongqing, and the middle reaches of the Yangtze River. To enrich the research on the assessment of the eco-efficiency of urban agglomerations, this paper evaluates the eco-efficiency levels of these urban agglomerations from 2006 to 2020. By making a longitudinal and horizontal comparative analysis of their spatiotemporal differences and evolution trends to realize the assessment of urban agglomeration ecological efficiency. In addition, based on exploring the spatial differences and distribution dynamics of urban eco-efficiency, the paper utilizes Geodetector to identify the internal and external influencing factors of urban eco-efficiency and the mechanism of spatial pattern formation to provide a basis for the differentiated improvement of urban agglomeration eco-efficiency. Thus, the paper reveals the characteristics of eco-efficiency from two different perspectives: a spatiotemporal perspective and a global-local perspective.

The main innovation of this paper is the in-depth comparison of the urban eco-efficiency of the five major urban agglomerations in China, as well as the examination of their spatiotemporal differentiation characteristics and driving factors.

In contrast to studies that primarily focus on the eco-efficiency performance of specific urban agglomerations [25, 26, 28, 29], this paper aims to explore the differences in eco-efficiency levels among different urban agglomerations, identify key factors that influence these variations, and conduct a longitudinal comparative analysis across different periods. Considering the substantial disparities in the development levels of various urban agglomerations in China, the conclusions and recommendations derived from this analysis are expected to provide more insightful insights. These innovations provide theoretical support and policy suggestions for the policy formulation of ecological civilization construction and regional green and coordinated development of urban agglomerations in China.

The rest of the paper is organized as follows. Section 3 details the methodologies used for assessing eco-efficiency and the method of Geodetector used for determining the influencing factors on eco-efficiency. Section 4 provides a detailed introduction to indicator selection and data source. Further, the results of the eco-efficiency and exploring the influencing factors of eco-efficiency are discussed in detail in Section 5. The final section details the conclusions and their implications for policymaking.

## 3. Methodology

There are three main parts in the research methodology of this paper. First, the DEA model considering the linkage of desirable output and undesirable output is used to measure the urban eco-efficiency of five major urban agglomerations. Second, the Theil Index is introduced to decompose the obtained eco-efficiency result and analyze the spatial-temporal differentiation of urban eco-efficiency and its sources. Third, the Geodetector is used to explore and analyze the driving factors of eco-efficiency differences in urban agglomerations as a whole and within each urban agglomeration.

### 3.1. Urban eco-efficiency evaluation model

In this section, we propose an evaluation model of urban eco-efficiency based on the USBM model which was developed by Tone (2001) [21]. The model is a non-radial and non-angular DEA model based on slack variables. It assumes that there is no linkage between input factors, desirable output factors, and undesirable output factors. However, in the economic environment system, the environmental factors as undesirable output are often the concomitants of the desirable output (economic development). In other words, there is an obvious correlation between them, which must obey certain quantitative relationships and satisfy specific production laws. Therefore, based on this model, a more reasonable eco-efficiency evaluation model is constructed by considering the linkage of the desirable and undesirable outputs. The urban eco-efficiency evaluation model that meets the requirements of the coordinated development of economic and environmental systems is constructed as follows:

$$min\rho =\frac{1-\frac{1}{m_{0}}[\sum_{i=1}^{m_{01}}\frac{S_{j}^{s-}}{x_{ik}^{s}}+m_{02}(1-\alpha_{k})+\sum_{i=1}^{m_{02}}\frac{S_{j}^{ns-}}{x_{ik}^{ns}}]}{1+\frac{1}{m_{1}+m_{2}}[\sum_{r=1}^{m_{11}}\frac{S_{r}^{sg+}}{y_{ik}^{sg}}+m_{12}(1-\alpha_{k})+\sum_{r=1}^{m_{12}}\frac{S_{r}^{nsg+}}{y_{rk}^{nsg}}+\sum_{l=1}^{m_{21}}\frac{S_{l}^{sb-}}{y_{lk}^{sb}}+m_{22}(1-\alpha_{k})+\sum_{l=1}^{m_{22}}\frac{S_{l}^{nsb-}}{y_{lk}^{nsb}}]},$$

$$s.t.\left\{ \begin{array}{c} x_{k}^{s}=X^{s}\lambda +S^{s-}\\ {\alpha_{k}x}_{k}^{ns}=X^{Ns}\lambda +S^{ns-}\\ y_{k}^{sg}=Y^{sg}\lambda -S^{sg+}\\ {\alpha_{k}y}_{k}^{nsg}=Y^{nsg}\lambda -S^{nsg+}\\ y_{k}^{sb}=Y^{sb}\lambda +S^{sb-}\\ {\alpha_{k}y}_{k}^{nsb}=Y^{nsb}\lambda +S^{nsb-}\\ 0\leq \alpha \leq 1\\ \lambda ,s^{s-},s^{ns-},s^{sg+},s^{nsg+},s^{sb-},s^{nsb-}\geq 0 \end{array}\right.$$ (1)

Where *ρ* is the level of urban ecological efficiency; *m*_0_, *m*_1_, *m*_2_ represent the number of input factors, desirable output, and undesirable output, respectively. Input factor *m*_0_ is further decomposed into separable input *m*_01_ and non-separable input *m*_02_, which satisfies the equation *m*_0_ = *m*_01_+*m*_02_. Desirable output *m*_1_ is decomposed into separable desirable output *m*_11_ and non-separable desirable output *m*_12_, which satisfies the equation *m*_1_ = *m*_11_+*m*_12_. Undesirable output *m*_2_ is decomposed into separable undesirable output *m*_21_ and non-separable undesirable output *m*_22_, which satisfies the equation *m*_2_ = *m*_21_+*m*_22_.

Based on the current research results [21, 28, 32], this paper assumes that the input factors of urban ecological efficiency evaluation mainly include labor input, land input, capital input, and energy input, while the desirable output is GDP. The undesirable output mainly includes environmental pollution variables such as carbon emissions, SO2 emissions, and wastewater. Additionally, the generation of exhaust gas and energy input are closely related and cannot be separated. In Eq (1), variables *x*, *y*, and *s* represent the input variables, the output variables, and the slack variables, respectively. The superscripts *s* and *ns* are used to distinguish the separable and non-separable variables. Specifically, *s* denotes the separable variables and *ns* denotes the non-separable variables. Thus, the separable and non-separable input vectors of the *kth* decision unit are expressed as $x_{k}^{s}$ and $x_{k}^{ns}$, respectively; the separable and non-separable desirable output vectors are expressed as $y_{k}^{sg}$ and $y_{k}^{nsg}$, respectively; the separable and non-separable undesirable output vectors are expressed as $y_{k}^{sb}$ and $y_{k}^{nsb}$, respectively. *s*^*s*−^ and *s*^*ns*−^, *s*^*sg*+^ and *s*^*nsg*+^, *s*^*sb*−^ and *s*^*nsb*−^ represent the variable matrices of separable and non-separable input slack variables, desirable output slack variables, and undesirable output slack variables, respectively. To simplify the model, the non-separable individual elements are assumed to vary in the same proportion α.

### 3.2. Theil Index

The Theil index was first proposed by Theil in 1967. Theil Index calculates the status of income inequality based on the concept of entropy in information theory which is suitable for measuring the differences and sources of high-quality development in different regions [33]. The advantage of the Theil index is that it can decompose the regional differences into two parts: intra-region and inter-region. This is conducive to further evaluating the contribution rate of inter-region and intra-regional differences to the overall regional differences.

In this study, urban agglomerations are taken as regions and the cities as the base units, while the urban samples are divided into multi-level structures. Based on the decomposability of the Theil Index, the overall regional differences can be decomposed into two parts: intra-regional and inter-regional differences. The contributions of different parts in the overall regional differences can be obtained to identify the internal structure and the reason for regional differences. Theil Index is calculated as follows:

$$T=\frac{1}{n}\sum_{i=1}^{n}\frac{Y_{i}}{y}\ln\left(\frac{Y_{i}}{y}\right).$$ (2)

Where *T* is the Thiel Index of high-quality development; *Y*_*i*_ is the level of quality development of the region *i* and is defined as the eco-efficiency value of the region *i* in this research; *y* denotes the average level of regional high-quality development, i.e., the average eco-efficiency value of all urban samples. We assume *T*∈[0,1]. The smaller the value, the smaller the regional difference.

There are *n* city samples divided into *K* regions. The number of cities in the region *k* is *n*_*k*_, and we have $\sum_{k=1}^{K}n_{k}=n$. Moreover, *Tb* and *Tω* denote the intra-regional differences and inter-regional differences of the Theil Index, respectively. The overall regional difference *T* can be decomposed into two parts, as shown in the following equation:

$$T=Tb+T\omega =\sum_{k=1}^{K}y_{k}ln(\frac{y_{k}}{n_{k}/n})+\sum_{k=1}^{K}y_{k}(\sum_{i\in g_{k}}\frac{y_{i}}{y_{k}}ln\frac{{y_{i}/y}_{k}}{1/n_{k}}).$$ (3)

Where *y*_*k*_ represents the ratio of the eco-efficiency value of region *k* to the sum of the eco-efficiency values of all samples; *y*_*i*_ represents the ratio of the eco-efficiency value of city *i* in region *k* to the sum of the eco-efficiency values of all samples.

### 3.3. Geodetector

GeoDetector is a statistical method to detect temporal-spatial heterogeneity [34, 35]. We employ Geodetector to explore the driving factors affecting the spatiotemporal evolution of urban eco-efficiency from the perspective of urban agglomerations which is commonly used in regional economic analysis. The core idea of Geodetector is that if independent variable X significantly influences dependent variable Y, then the distribution of the independent variable and the dependent variable should be similar. It is assumed that the areas selected in this research are divided into several strata (or regions). If the sum of the variances of each stratum (or region) is less than the total variance, then there is spatial heterogeneity in the study object. Geodetector is an important tool for analyzing spatial heterogeneity and exploring the relative importance and strength of the interaction of driving factors. In this paper, Geodetector is used to investigate the influence degree of various factors on the urban eco-efficiency of urban agglomerations. The independent variable X is the selected indirect index affecting urban eco-efficiency, while the dependent variable Y is the value of the urban agglomeration eco-efficiency. The formula of Geodetector is

$$q=1-\frac{1}{N\sigma^{2}}\sum_{n=1}^{L}N_{h}\sigma_{h}^{2}.$$ (4)

where *q* represents the determining force of the driving factor. We have *q*∈[0,1]. A larger value of *q* indicates a more obvious spatial heterogeneity of the dependent variable Y. Since the stratification is generated by the independent variable X, the larger the value of *q*, the stronger the explanatory power of influencing factor X to urban eco-efficiency Y, and vice versa. In extreme cases, *q* = 1 indicates that the spatial distribution of Y is completely determined by factor X, while *q* = 0 indicates that X and Y are irrelevant. Additionally, *h* is the number of strata (or regions) of the independent variable, which in this study is the type of factors influencing the spatial differences of eco-efficiency in different stages of each urban agglomeration. Moreover, *N* and *N*_*h*_ are the total number of samples and the sample size of strata *h*, respectively; *σ*^2^ and $\sigma_{h}^{2}$ denote the variance of Y values in the overall and strata *h*, respectively.

## 4. Study area and data source

### 4.1. Study area

According to the current distribution of urban agglomerations in China and existing research results, the Yangtze River Delta urban agglomeration, Pearl River Delta urban agglomeration, Beijing-Tianjin-Hebei urban agglomeration, Chengdu-Chongqing urban agglomeration, and middle reaches of the Yangtze River urban agglomeration are selected as the research objects, including 93 cities. The specific distribution is shown in Table 1.

**Table 1:** Composition of five major urban agglomerations in China.

Urban agglomerations	Cities
Beijing-Tianjin-Hebei	Beijing, Tianjin, Shijiazhuang, Tangshan, Qinhuangdao, Handan, Xingtai, Baoding, Zhangjiakou, Chengde, Cangzhou, Langfang, Hengshui
Chengdu-Chongqing	Chongqing, Chengdu, Zigong, Luzhou, Deyang, Mianyang, Suining, Neijiang, Leshan, Nanchong, Meishan, Yibin, Guang ’an, Dazhou, Ya ’an, Ziyang
Middle Reaches of Yangtze River	Nanchang, Jingdezhen, Pingxiang, Jiujiang, Xinyu, Yingtan, Ji ’an, Yichun, Fuzhou, Shangrao, Wuhan, Huangshi, Yichang, Xiangfan, Ezhou, Jingmen, Xiaogan, Jingzhou, Huanggang, Xianning, Changsha, Zhuzhou, Xiangtan, Hengyang, Yueyang, Changde, Yiyang, Loudi
Yangtze River Delta	Shanghai, Nanjing, Wuxi, Changzhou, Suzhou, Nantong, Yancheng, Yangzhou, Zhenjiang, Taizhou, Hangzhou, Ningbo, Wenzhou, Jiaxing, Huzhou, Shaoxing, Jinhua, Zhoushan, Taizhou, Hefei, Wuhu, Maanshan, Tongling, Anqing, Chuzhou, Chizhou, Xuancheng
Pearl River Delta	Guangzhou, Shenzhen, Zhuhai, Foshan, Jiangmen, Zhaoqing, Huizhou, Dongguan, Zhongshan

### 4.2. Variables selection and data source

To measure eco-efficiency comprehensively and accurately, one desirable output, three undesirable outputs, and four inputs required for the eco-efficiency evaluation component of this study are shown in Table 2.

**Table 2:** Input and output indicator.

Category	Item	Indicator
Desirable output	Economic aggregate	GDP
Undesirable output	Exhaust gas emission	Industrial SO₂ emission (SO2)
	Wastewater discharge	Industrial wastewater discharge (wastewater)
	Carbon dioxide emissions	Total carbon dioxide emissions (CO2)
Input	Labor input	Number of employees in each city unit at the end of the period (labor)
	Land input	City construction land area (land)
	Capital input	Fixed capital stock (capital)
	Energy input	Industrial electricity consumption (electricity consumption)

Specifically, the capital input is estimated by adopting the Perpetual Inventory Method [36, 37]. The number of employed persons was usually taken as the proxy variable of labor input. Following the extant literature, we used the amount of electricity consumption as a proxy for energy input due to the lack of data on final energy consumption at the city level [38]. Regarding the land input, we took construction land as the proxy variable [39]. The desirable output involved in the efficiency evaluation model is denoted by Gross Domestic Product (GDP) in 2005 constant prices. Regarding the undesirable outputs, we in this study considered three important pollutants, namely wastewater, SO2, and CO2 due to data availability at city levels. Chen et al. (2020) [6] and Zhang et al. (2021) [27] considered the industrial smoke (powder) dust as an unexpected output. However, with the proposal of China’s carbon neutrality and peak carbon target, the Chinese government has established and emphasized mandatory reductions in the three pollutants in the Five-Year Plans (wastewater, SO2, and CO2), which are the most representative [40].

To further explore the spatial heterogeneity of urban eco-efficiency and its driving factors, the industrial structure (proportion of secondary industry), energy structure, innovation level, fiscal level, Foreign direct investment(FDI) proportion, and population density are selected as indirect indicators affecting urban eco-efficiency, considering the existing studies and the applicability of city-level indicators [28, 41].

Specifically, extant papers have confirmed that industrial structure, especially the secondary industrial could affect the eco-efficiency. We use the shares of the total output of the secondary industry in GDP as the proxy [41]. The relationship between FDI and green development is uncertain. The advanced technologies that come with FDI could help to promote economic development, but resource consumption and pollution emissions might also increase as a result of FDI and thus have a negative impact [29]. We use the shares of foreign direct investment (FDI) in GDP as the proxy. Innovation plays a crucial role in reducing pollutants and increasing eco-efficiency [28, 29]. In this study, we utilize the innovation index, proposed by Kou and Liu (2017) [42], to measure the innovation capacities of Chinese cities. In addition, environmental infrastructure investment is related to environmental construction and pollution improvement and is an important tool for environmental governance [31]. The impact of indicators such as environmental pollution control investments, environmental infrastructure investments, environmental infrastructure investments per capita, and environmental pollution control investments per capita on urban eco-efficiency are also considered in this paper. The GeoDetector tool is used to detect the influence of each factor on the eco-efficiency of the cities. On this basis, the dominant factors of spatial differences in eco-efficiency of the total sample of the cities and the samples of the five major urban agglomerations in China are revealed.

Based on the method proposed by Wu (2008) [43], we use the inter-provincial capital stock data to estimate the urban capital stock data, while carbon emission data are obtained from the Carbon Emission Accounts& Datasets (CEADs).

The research data above are derived from the China City Statistical Yearbook (2007–2021), China Environmental Yearbook (2007–2021), and China Statistical Yearbook For Regional Economics (2007–2021). Some data are supplemented according to the Statistical Yearbook of Provinces (municipalities) and the Statistical Communique of National Economic and Social Development of relevant municipalities. The interpolation method is adopted to calculate the missing data according to the actual situation. The municipal administrative unit is the minimum decision-making unit in space.

## 5. Empirical results and discussions

### 5.1. Results of eco-efficiency

**Fig 1:**
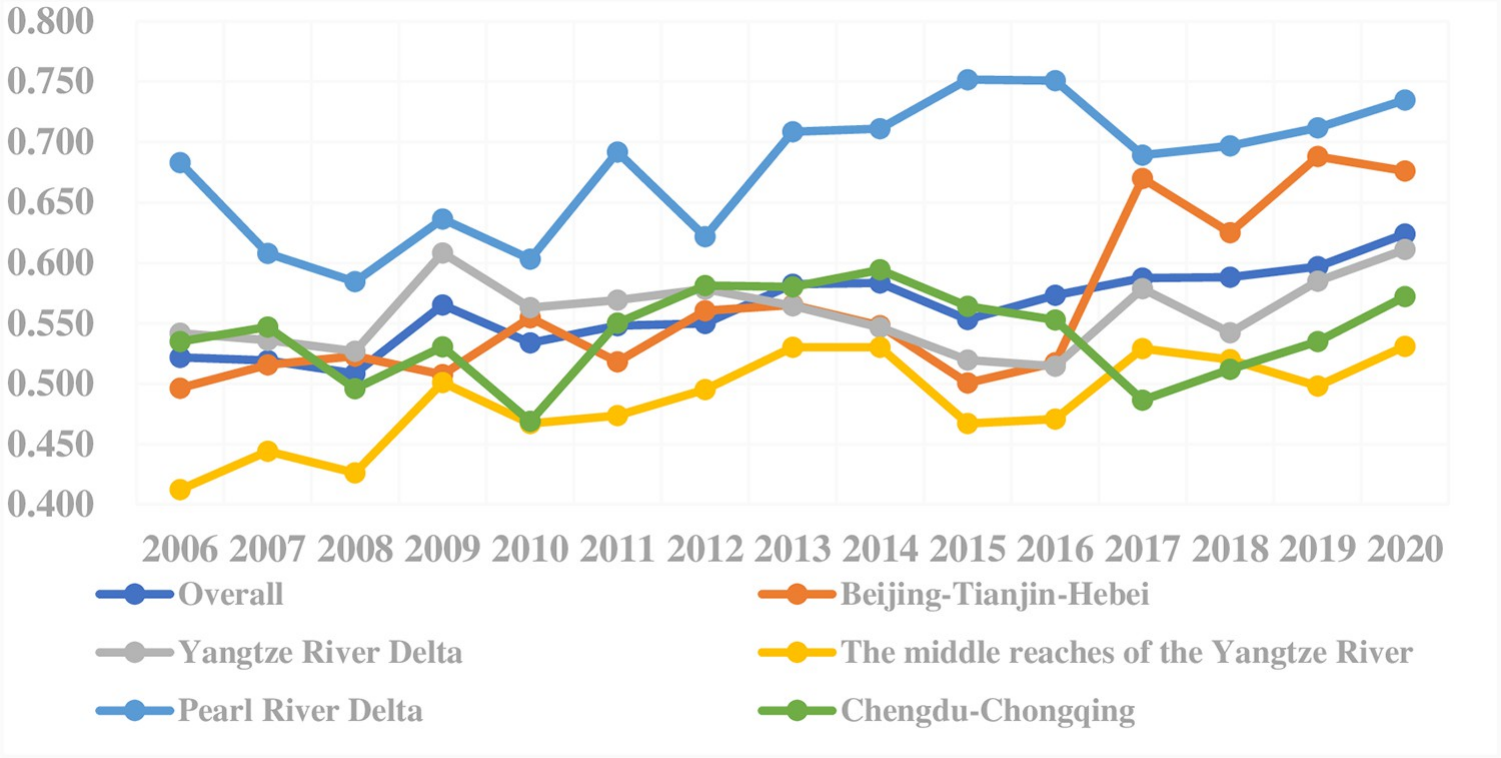
Average eco-efficiency of five major urban agglomerations in China from 2006 to 2020.

Referring to the model mentioned in 2.1, this study assumes that among the input and output factors of the model, industrial electricity consumption and industrial SO2 emissions are inseparable, while other factors are separable. According to the eco-efficiency evaluation model, the mean eco-efficiency of the whole sample of cities and the five major urban agglomerations from 2006 to 2020 can be calculated, as shown in Fig 1.

In terms of annual mean changes, the mean eco-efficiency values of all the cities showed a fluctuating trend of rising from 2006 to 2020. Comparing the eco-efficiency levels of the full sample of cities with those of the five major urban agglomerations, the eco-efficiency levels of the city agglomerations in the middle reaches of the Yangtze River, Chengdu-Chongqing, and the Yangtze River Delta are all lower than the national average (the eco-efficiency value of China). The mean eco-efficiency of major urban agglomerations showed a significant increase in 2009 compared with 2008. A similar situation also appeared in 2017. In 2009, environmental pollution control was proposed as a major strategic decision deployment for the first time. In 2017, the eco-efficiency of other urban agglomerations increased significantly, but that of the Pearl River Delta urban agglomeration declined significantly. According to the “Urban Ambient Air Quality Situation in Guangdong Province” (2017, 2016), the average proportion of days on which the air quality index (AQI) reached the standard in the Pearl River Delta urban agglomeration was 84.5% in 2017, which shows a significant decline compared to the average value of 89.5% in 2016. After merging with Jianyang in 2016, Chengdu announced in early 2017 that its central urban area would be expanded from the previous "5+1" to a new "11+2" metropolitan pattern. The continuous expansion and extensive development of Chengdu, are the contributing factors to the persistent decline in urban eco-efficiency within the Chengdu-Chongqing urban agglomeration [8].

After 2018, the eco-efficiency values of the national average as well as most urban agglomerations, except for the middle reaches of the Yangtze River, have shown an upward trend. In 2018, the highest governmental leading department of eco-environmental governance in China, the Ministry of Environmental Protection (MEP), was officially renamed the " Ministry of Ecology and Environment (MEE)", which implies that the environmental governance and protection in China is more ecologically oriented. In the same year, the government also issued a series of policy documents to support ecological and environmental governance. For example, the State Council issued the "Opinions on Strengthening Ecological and Environmental Protection in All-Round Way and Firmly Fighting the Battle of Pollution Prevention and Control", which emphasizes that ecology flourishment is the sufficient condition of civilization flourishment. The degree of importance attached to ecology has been increased unprecedentedly.

From the perspective of different regions, the eco-efficiency value of the Pearl River Delta urban agglomeration always ranked first among the major urban agglomerations. Especially between 2012 and 2016, its advantage gradually expanded, which is closely related to the good economic development trend and environmental foundation of the Pearl River Delta [28, 29]. However, the eco-efficiency lead of the Pearl River Delta has dwindled since 2017.

The urban eco-efficiency of the middle reaches of the Yangtze River urban agglomeration was always in a relatively lagging position, while its development trend in recent years was consistent with the overall trend of the whole sample cities nationwide. Tone (2001) [21]found that the eco-efficiency levels of coastal cities in eastern China are significantly higher than those of inland cities. Among inland cities, the improvement in urban eco-efficiency levels is least prominent in the central region.

It should be noted that the average eco-efficiency value of the Yangtze River Delta urban agglomerations has been lower than the national average since 2013, while the mean value of the Beijing-Tianjin-Hebei and Chengdu-Chongqing urban agglomerations was lower than the national average level in most time of the sample period, which indicates that the eco-efficiency performance of different urban agglomerations in China shows prominent stratification characteristics. These findings illustrate the significant disparities in development among different urban agglomerations and cities within these agglomerations in China. Therefore, it is urgent to promote the coordinated development of urban agglomerations.

To further evaluate the performance of urban eco-efficiency within the five major urban agglomerations at the national level, we calculated the national average eco-efficiency level for all cities and compared the eco-efficiency value of each city with this national average. For a particular city, if its eco-efficiency value exceeds the national average in one year, it is classified as a high-quality development city. By contrast, low-quality development cities are those whose eco-efficiency values rank within the bottom 30% of all cities nationwide.

**Fig 2:**
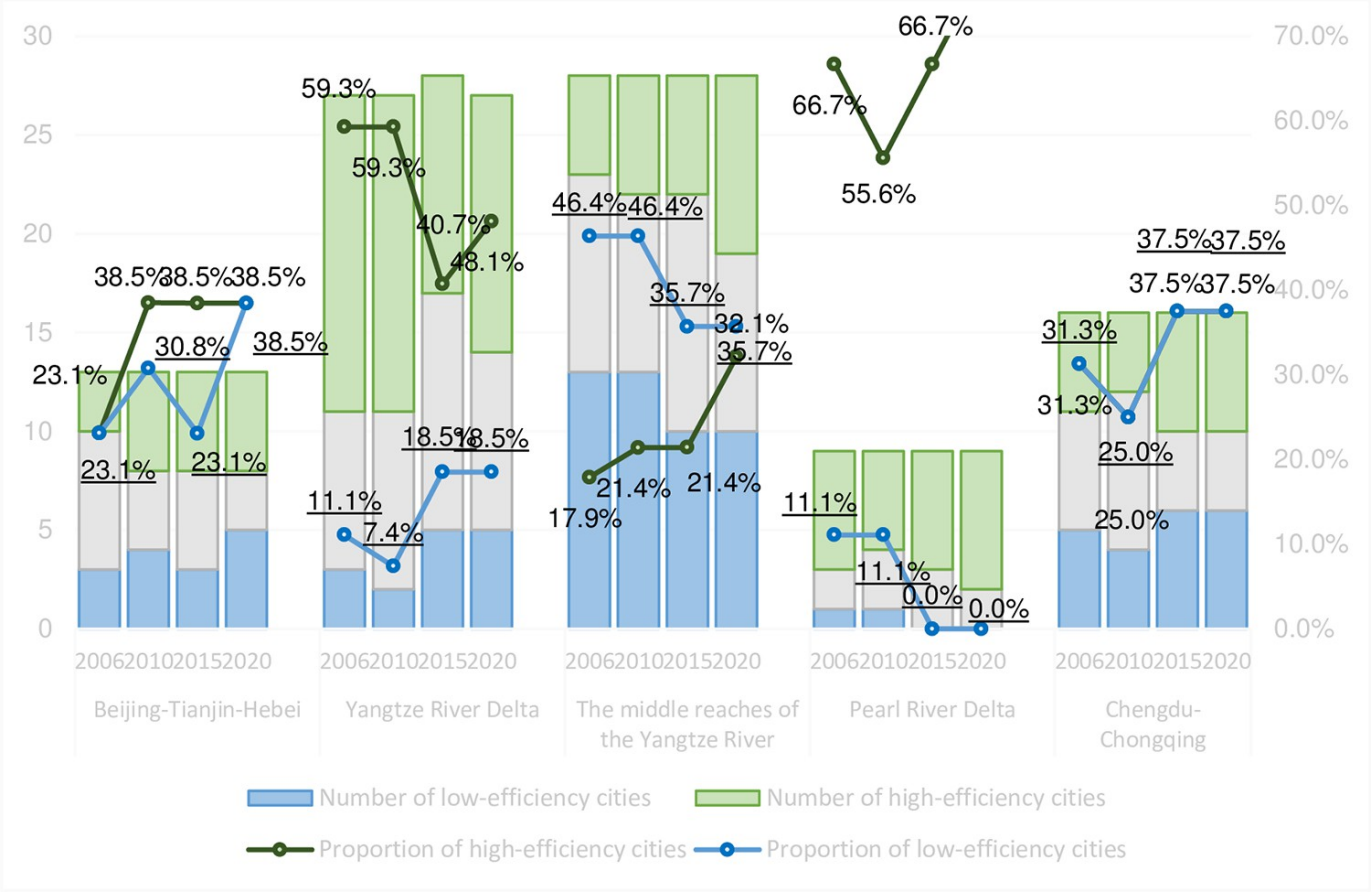
Number of high-efficiency and low-efficiency cities in urban agglomerations and their proportions.

According to the basic ideas of the Five-Year Plan for the national economy of China. Considering the years 2010, 2015, and 2020 were the last years of China’s 11th Five Year Plan, 12th Five Year Plan, and 13th Five Year Plan, respectively, we choose 2006 as the starting year of this study and summarize the statistical results of the high-quality and low-quality development cities in each urban agglomeration in 2006, 2010, 2014, and the last year of the sample period (2020), respectively. The results are shown in Fig 2.

On the whole, the number of high eco-efficiency cities accounted for 33.5% in 2006, 34.2% in 2010, 35.3% in 2015, and 36.0% in 2020, respectively. The number of high eco-efficiency cities increased slightly in each period, but the total number of high and low eco-efficiency cities has been relatively stable in recent years. As can be seen in Fig 2, the structure of the five major urban agglomerations differs significantly. The number of both high and low-eco-efficiency cities in the Beijing-Tianjin-Hebei urban agglomeration was on the rise, indicating a more pronounced polarization within this urban agglomeration. This is related to the result of polluting industries that have been transferred from Beijing to neighboring provinces and cities such as Hebei within the urban agglomeration.

While the number of high eco-efficiency cities in the Yangtze River Delta urban agglomeration and Chengdu-Chongqing urban agglomeration declined, the number of low eco-efficient cities increased, indicating that there are some problems in eco-efficiency within the region that need to be taken seriously. In the middle reaches of the Yangtze River urban agglomeration, the number of high eco-efficiency cities increased slightly and that of low eco-efficiency cities decreased significantly, indicating that although the eco-efficiency level of this urban agglomeration was not high, it was relatively more balanced within the region. Huang et al. (2018b) [2] indicated that among the three major urban agglomerations in the Yangtze River Economic Belt (Yangtze River Delta, middle reaches of the Yangtze River, and Chengdu-Chongqing), the middle reaches of the Yangtze River urban agglomeration exhibits a more pronounced clustering effect in terms of eco-efficiency improvement. Among the five major urban agglomerations, the Pearl River Delta urban agglomeration had the highest proportion of high eco-efficiency cities, showing an excellent green economic foundation. Zhou et al. (2018) [28] attribute this reason to relatively loose government regulations, high levels of openness, and high population density.

### 5.2. Results of Theil Index

**Fig 3:**
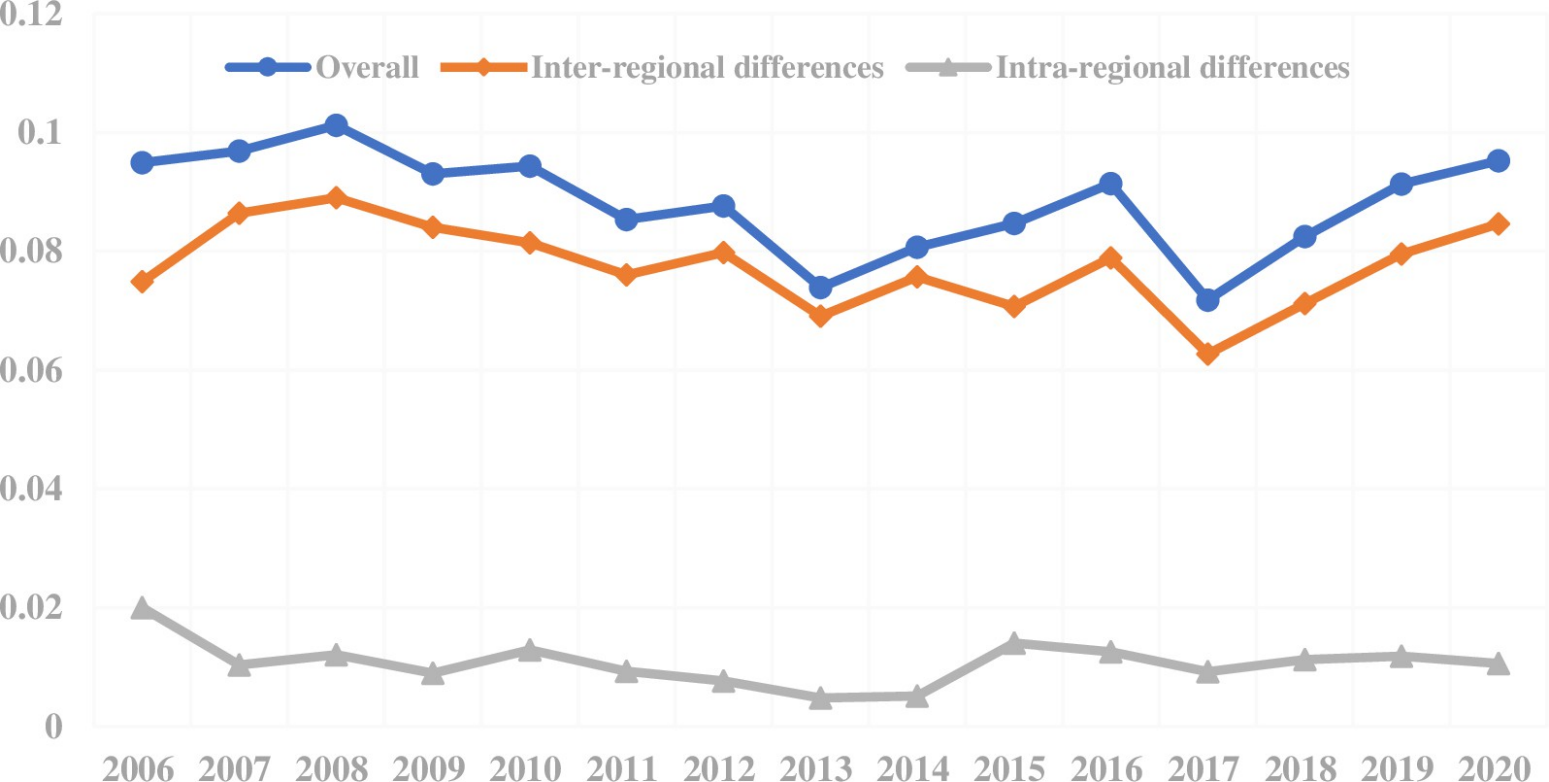
Spatial differences in eco-efficiency in five major urban agglomerations in China from 2006 to 2020.

Considering the vast territory of China and the large differences between different urban agglomerations in terms of natural conditions, resource endowments, and industrial structures, the impact of spatial heterogeneity cannot be ignored in the eco-efficiency evaluation. Therefore, in this paper, *Tb* and *Tω* obtained from the decomposition of the Theil Index represent the intra-regional and inter-regional differences in urban eco-efficiency, respectively. The specific results are shown in Fig 3.

The overall differences in the eco-efficiency of all sample cities showed a fluctuating downward trend. A declining trend was pronounced from 2008 to 2013, which showed good synergy and consistency in the development of eco-efficiency levels across cities in China. In terms of specific time points, the difference between 2013 and 2017 showed an obvious drop, because 2013 and 2017 happened to be critical years when China put great emphasis on ecological and environmental protection. The overall, intra-regional, and inter-regional differences increased slightly in 2017. Since this year, China has continuously strengthened its emphasis on environmental protection and regional coordinated development, which has also prompted major cities to attach importance to eco-efficiency and promoted the improvement of the synergy and consistency of eco-efficiency levels among cities across the country. The "National Ecological Conservation 12th Five-Year Plan" and the "Ecological and Environmental Protection 13th Five-Year Plan" were released and implemented in China in January 2013 and November 2016, respectively. These two documents serve as the guiding principles for ecological and environmental protection in China. They are taken more seriously by local governments. As a result, the eco-efficiency scores have been gradually increasing since 2013 [29]. Huang et al. (2018c) [38] also obtained similar results in the study of the middle reaches of the Yangtze River urban agglomeration, Chengdu-Chongqing urban agglomeration, and Yangtze River Delta urban agglomeration in which the eco-efficiency level in 2013 was even lower than that in 2003.

Further observation of intra-regional and inter-regional differences obtained from the decomposition of overall differences shows that the contribution of inter-regional differences has been small. It is the intra-regional differences that affect the level of differentiation of eco-efficiency in different cities. However, since 2015, although the contribution of inter-regional differences still showed a decreasing trend, the contribution of inter-regional differences has increased compared with that in the previous period, which indicates that the performance of eco-efficiency levels of cities in different regions began to diverge. Moreover, the difference in eco-efficiency within urban agglomerations has been improved, which is consistent with the fact that environmental pollution control actions are mainly implemented separately based on local interests.

**Fig 4:**
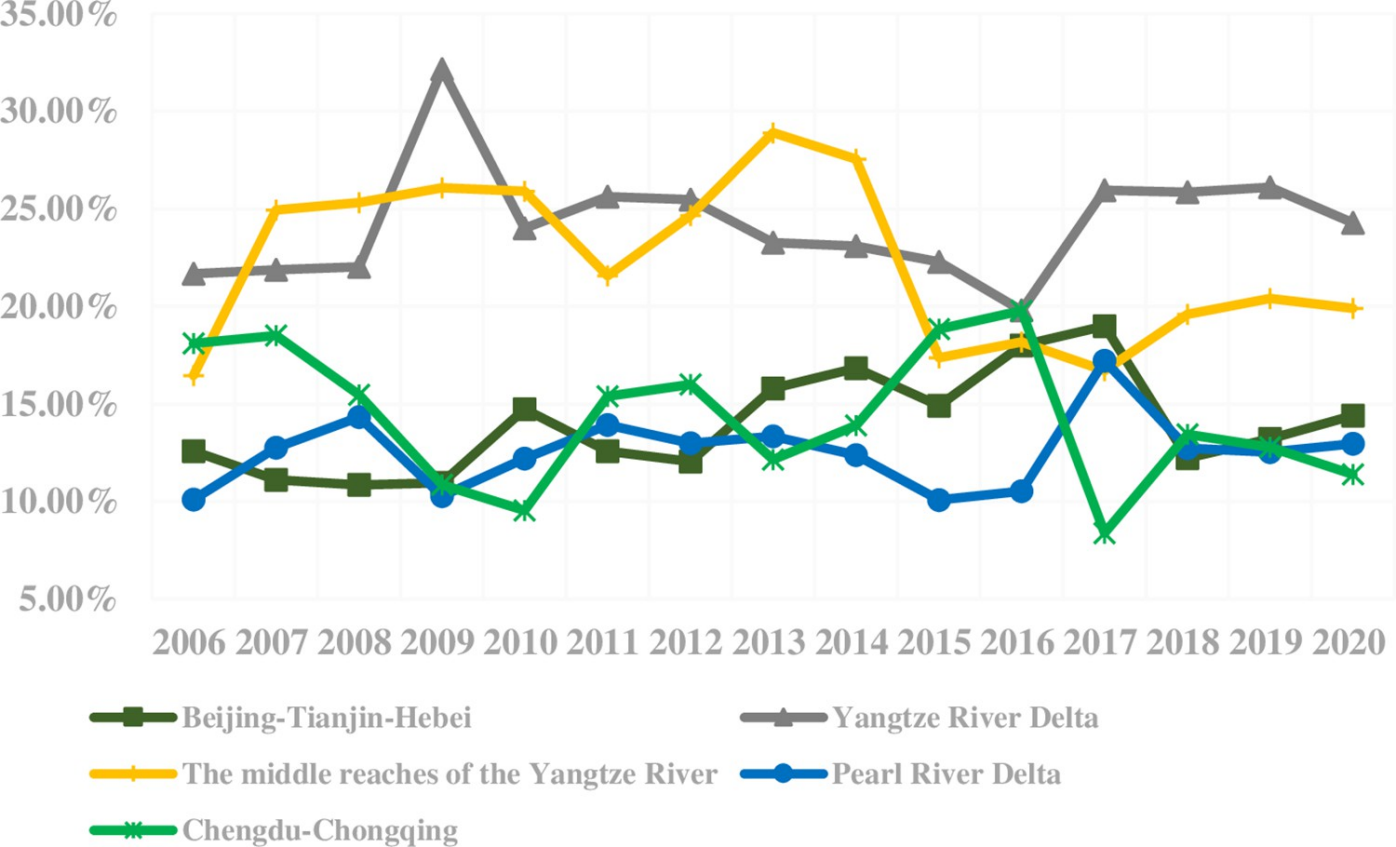
Contribution degree of eco-efficiency differences of five major agglomerations to the overall difference of all sample cities in China.

The *T* value calculated by the Theil Index represents the contribution of eco-efficiency differences of the urban agglomerations to the overall difference of all sample cities in China. The result is shown in Fig 4. During the inspection period, the contribution of the different degrees of the Yangtze River Delta urban agglomeration was continuously higher than the other urban agglomerations, while the contribution degree of the Pearl River Delta urban agglomeration was continuously lower. Since 2013, the contribution of the middle reaches of the Yangtze River urban agglomeration decreased significantly, while that of the Beijing-Tianjin-Hebei urban agglomeration showed a trend of increasing. By contrast, the volatility of the contribution to differences in the Chengdu-Chongqing urban agglomeration was significantly larger.

### 5.3. Influencing factors of eco-efficiency

In general, the pursuit of improving eco-efficiency aims to achieve a win-win situation for the economy, society, and the ecological environment. According to the research results above, there are obvious differences in eco-efficiency performance among the urban agglomerations, which can be attributed to the economic and social development differences among urban agglomerations. Combining the existing studies and the applicability of city-level indicators [32, 33], GeoDetector is used to detect the determining force of various factors mentioned above, and then to reveal the dominant factors of spatial differences in eco-efficiency of the whole sample cities and the five major urban agglomerations in China. Considering that China’s economy develops in an orderly manner according to the five-year plan, and the 13th Five-Year Plan for ecological and environmental protection which started in 2016 and considers the environment as a binding indicator for local development for the first time, we further decompose the analysis of the influencing factors into three stages. The first stage (2006–2010) is the 11th Five Year Plan period in China, the second stage (2011–2015) is the 12th Five Year Plan period in China, and the third stage (2016–2020) is the 13th Five Year Plan period in China. The results of driving factors *q* obtained at different stages in each region by GeoDetector are shown in Table 3.

**Table 3:** Influencing factors of spatial difference in eco-efficiency at different stages in each urban agglomeration.

Years	Factors	Overall	Beijing-Tianjin-Hebei	Yangtze River Delta	Middle reaches of Yangtze River	Pearl River Delta	Chengdu-Chongqing
2006–2010	Proportion of secondary industry	0.058*	0.722*	0.345*	0.216**	0.521*	0.271*
2011–2015		0.118***	0.601*	0.55**	0.326**	0.802*	0.413**
2016–2020		0.072***	0.132*	0.356**	0.243**	0.423*	0.256**
2006–2010	Energy structure	0.075**	0.544***	0.548**	0.525**	0.592**	0.107**
2011–2015		0.044*	0.639***	0.352**	0.404**	0.533**	0.536*
2016–2020		0.042**	0.523**	0.211**	0.201**	0.541*	0.254**
2006–2010	Innovation level	0.112**	0.669**	0.659****	0.374***	0.726***	0.458***
2011–2015		0.17***	0.662**	0.19***	0.201***	0.741***	0.374**
2016–2020		0.262***	0.361***	0.122***	0.21***	0.189***	0.048***
2006–2010	Fiscal level	0.065	0.634	0.253*	0.192**	0.504***	0.339*
2011–2015		0.038*	0.745**	0.048*	0.362**	0.138***	0.234*
2016–2020		0.103*	0.425*	0.052*	0.375**	0.528**	0.375*
2006–2010	FDI proportion	0.071**	0.27***	0.159***	0.215***	0.548***	0.686***
2011–2015		0.057**	0.966***	0.214***	0.07***	0.801*	0.186***
2016–2020		0.124*	0.425***	0.316***	0.125***	0.124*	0.416***
2006–2010	Population density	0.091***	0.612***	0.314	0.223*	0.539*	0.518*
2011–2015		0.121***	0.606*	0.129*	0.266*	0.048*	0.538*
2016–2020		0.158**	0.768**	0.219**	0.462*	0.215**	0.364*
2006–2010	Environmental pollution control investment	0.202***	0.487**	0.582***	0.455***	0.970**	0.235***
2011–2015		0.164***	0.452**	0.178**	0.144***	0.812***	0.375***
2016–2020		0.312***	0.421**	0.512**	0.312***	0.712***	0.261***
2006–2010	Environmental infrastructure investment	0.196***	0.365**	0.546**	0.355***	0.851***	0.235**
2011–2015		0.134**	0.374***	0.155**	0.096***	0.802**	0.371**
2016–2020		0.216*	0.561***	0.312**	0.217**	0.513***	0.232**

*, **, and *** indicate it’s significant at the 10%, 5%, and 1% significance levels, respectively.

From the perspective of the whole sample, the top three factors influencing the spatial distribution difference of urban eco-efficiency were the environmental pollution control investments, innovation level, and environmental infrastructure investments. All the factors have passed the 1% significance test. Specifically, the environmental pollution control investments and the environmental infrastructure investments represent the level of government attention to environmental pollution control. Moreover, both the environmental pollution control investments and the environmental infrastructure investments are the strength of government finances, which play an important and positive role in improving the governance effectiveness of China’s environment [44].

The attention and technical support of the government are the key factors to improve and enhance urban eco-efficiency. Sufficient fiscal support can promote green technological innovation to improve eco-efficiency because fiscal policies can effectively correct the failure of market mechanisms in long-term and large-scale investments [45]. The difference from the conclusions of the current literature is that the contribution levels of both energy structure and FDI proportion to the spatial heterogeneity of urban eco-efficiency in the whole sample were relatively low. Tong et al. (2021) [29] explored the effects of FDI on the eco-efficiency of Chinese cities from 2005 to 2016, indicating that the effect is a complex problem for the results depending on different stages of economic development. among different regions. The former indicates that although the wide variation in energy structure exists in different regions of China, the role of energy structure endowment on urban eco-efficiency improvement is not significant under the overall arrangement of the national energy package. The latter indicates that the heterogeneity of the “pollution paradise hypothesis” in Chinese cities is not obvious.

As can be seen in Table 3, from the perspective of urban agglomerations, the factors influencing the spatial distribution difference of urban eco-efficiency are different. For the Beijing-Tianjin-Hebei and the middle reaches of the Yangtze River urban agglomeration, the top three influencing factors were population density, innovation level, and energy structure. The difference is that the FDI proportion had a more significant impact on the Beijing-Tianjin-Hebei urban agglomeration and a minor impact on the middle reaches of the Yangtze River urban agglomeration. The result shows once again the complexity of the effect of FDI on eco-efficiency [29]. The contribution of industrial structure (proportion of secondary industry) and environmental pollution control investments to the spatial difference of urban eco-efficiency in the Yangtze River Delta urban agglomeration and the Pearl River Delta urban agglomeration were both in the top two positions. In the horizontal comparison, the influence levels of FDI proportion and population density on the spatial difference of urban eco-efficiency in these two agglomerations were both in the last two places. These two agglomerations are both regions with higher population density and more developed export-oriented economies, which may imply that the influence of FDI proportion and population density may be overestimated. Among the five major urban agglomerations, the factors affecting the spatial distribution difference of urban eco-efficiency in Chengdu-Chongqing urban agglomeration are significantly different from those in other urban agglomerations. For the Chengdu-Chongqing, the top three contributors were population density, FDI proportion, and industrial structure (proportion of secondary industry). At the same time, environmental pollution control investment, innovation level, and environmental infrastructure investment, which had more apparent effects on other urban agglomerations, did not have significant effects on the Chengdu-Chongqing urban agglomeration, which may be closely related to the current economic development stage of Chengdu-Chongqing urban agglomeration and the industrial characteristics that are relatively more focused on the heavy industry [46].

From the perspective of time intervals, the impact of various factors on urban agglomerations varies significantly at different time stages. Overall, the influence degree of the proportion of secondary industry and energy structure on the urban eco-efficiency of all the urban agglomerations tended to decrease in all three periods, while the importance degree of fiscal level increased. For the Beijing-Tianjin-Hebei and Yangtze River Delta urban agglomeration, the importance of population density and environmental infrastructure investment showed a decreasing and then increasing trend, while the influence of environmental pollution control investment on the Yangtze River Delta was also increasing. Although the contribution of FDI proportion was not high, its influence increased significantly. For the middle reaches of the Yangtze River urban agglomeration, the influence degree of innovation level increased first and then decreased, while the influence degree of population density continued to rise. For the Pearl River Delta urban agglomeration, the impact of FDI proportion decreased significantly; the impact of population density increased significantly; the environmental pollution control investment and environmental infrastructure investment continued to decrease. For the Chengdu-Chongqing urban agglomeration, the impact of the fiscal level increased most significantly, while the innovation level had a significant decrease.

## 6. Conclusions and policy implications

Based on the eco-efficiency evaluation model, this paper investigates the urban eco-efficiency of five major urban agglomerations in China and analyzes the spatial-temporal differences in eco-efficiency and its driving factors. The empirical results are as follows.

1. In terms of eco-efficiency performance, the average level of eco-efficiency of the full sample of cities shows that the overall level of eco-efficiency of China’s cities has shown a fluctuating upward trend from 2006 to 2020, especially after 2015, when the growth trend is more obvious. Comparing the full sample of cities with the five major urban agglomeration, despite the obvious advantages of the urban agglomeration in terms of economic development level, the eco-efficiency level of the urban agglomeration in the middle reaches of the Yangtze River, Chengdu-Chongqing, and the Yangtze River Delta city clusters is lower than the national average level. From a structural point of view, the eco-efficiency performance of different urban agglomerations in China shows obvious non-stratified characteristics, and the eco-efficiency value of the PRD urban agglomeration has always been at the top of all major urban agglomerations, and the level of eco-efficiency has gradually increased. However, after 2017, the lead of the PRD urban agglomeration over other urban agglomerations in terms of eco-efficiency level has narrowed down, reflecting that the overall level of eco-efficiency of China’s cities is gradually improving. The urban eco-efficiency of the middle reaches of the Yangtze River urban agglomeration was always in a relatively backward position. By contrast, the mean values of eco-efficiency of the Yangtze River Delta, Beijing-Tianjin-Hebei, and Chengdu-Chongqing were lower than the national average most of the time. From the perspective of urban agglomeration, the proportion of high eco-efficiency cities was the highest in the Pearl River Delta urban agglomeration, and the polarization within the Beijing-Tianjin-Hebei urban agglomeration was evident. The eco-efficiency level of both high and low-eco-efficiency cities declined in the Yangtze River Delta urban agglomeration and the Chengdu-Chongqing urban agglomeration. Although the overall eco-efficiency level of the middle reaches of the Yangtze River urban agglomeration was not high, it was relatively balanced internally. From the perspective of input and output, the lack of desirable output and redundancy of undesirable output are the root causes of eco-inefficiency in urban agglomerations.
2. The overall differences in the urban eco-efficiency of all the sample cities showed a fluctuating downward trend, and the synergy and consistency of development of the urban eco-efficiency among the cities in China were good. The contribution of inter-regional differences was relatively small. It is the intra-regional differences that affect the level of differentiation of eco-efficiency in different cities. However, the contribution of inter-regional differences has increased since 2015, indicating that the performance of eco-efficiency levels of cities in different regions began to diverge. From the perspective of urban agglomeration, the contribution of regional differences of the Yangtze River Delta urban agglomeration was continuously at a relatively high level. On the contrary, the contribution of the Pearl River Delta urban agglomeration was continuously at a relatively low position. The contribution of regional differences in the middle reaches of the Yangtze River urban agglomeration decreased significantly, while the regional difference in the Beijing-Tianjin-Hebei urban agglomeration showed a fluctuating upward trend. The contribution of regional differences in Chengdu-Chongqing urban agglomeration fluctuated significantly.
3. From the perspective of driving factors, the top three factors affecting the spatial distribution difference of urban eco-efficiency in the whole sample were environmental pollution control investments, innovation level, and environmental infrastructure investments, which indicates that attention and technical support of the government are the key factors to improve the urban eco-efficiency. From the perspective of urban agglomerations, Beijing-Tianjin-Hebei and the middle reaches of the Yangtze River urban agglomeration should pay extra attention to the population density and energy structure. By contrast, the Yangtze River Delta and Pearl River Delta urban agglomeration should attach importance to the industrial structure (proportion of secondary industry). The Chengdu-Chongqing urban agglomeration needs to focus on the FDI proportion.

The results of the empirical analysis above have important implications for promoting the coordinated improvement of urban eco-efficiency in the urban agglomerations in China.

For one thing, to achieve coordinated improvement of eco-efficiency of urban agglomeration and narrow the gap of eco-efficiency level within urban agglomeration, different urban agglomerations should have different requirements for their internal cities. Therefore, the five urban agglomerations should focus on promoting the cooperative mechanism among cities in the field of environmental governance and prevention, and enhance the overall improvement of the eco-efficiency level of urban agglomerations by joint prevention and control. Several effective approaches include: establishing a regional joint prevention and control mechanism for ecological and environmental governance; making full use of the existing integrated network transportation system within the urban agglomerations; achieving the resource advantages complementary and information integration and interaction. Besides, each urban agglomeration should implement the strategy of improving eco-efficiency according to local conditions based on its weak points of eco-efficiency. The Pearl River Delta urban agglomeration should strive to promote the driving effect of high eco-efficiency cities on low eco-efficiency cities. The Beijing-Tianjin-Hebei urban agglomeration should focus on promoting the eco-efficiency level of low eco-efficiency cities to avoid polarization in the region. It is necessary to improve the eco-efficiency of all the cities in the middle reaches of the Yangtze River urban agglomeration. Both the Yangtze River Delta urban agglomeration and Chengdu-Chongqing urban agglomeration should pay more attention to the eco-efficiency level, and restrain the decline of the absolute level of high and low eco-efficient cities.

First of all, policymakers should support the strengths of high eco-efficiency cities while encouraging them to share their experiences and resources with low eco-efficiency cities, emphasizing the driving effect of high eco-efficiency cities on low eco-efficiency cities. Specifically, the policymakers of the Beijing-Tianjin-Hebei urban agglomeration should focus on the strict implementation of eco-environmental protection policies to make low eco-efficiency cities pay more attention to enhancing their eco-efficiency, thus avoiding polarization within the region. Meanwhile, it is recommended that Beijing set up special funds for eco-efficiency improvement, which should be used to courageously compensate and support other cities to improve their eco-efficiency level. The eco-efficiency of urban agglomeration in the middle reaches of the Yangtze River is at a low level, so policymakers should pay more attention to the dual goals of environmental governance and economic development from the perspective of setting and evaluating the governance goals, and support the cultivation of the demonstration cities in the urban agglomeration to take the lead in realizing the construction of high-efficiency eco-cities, thus realizing the goal of overall improvement of eco-efficiency level gradually. It is worth noting that the Yangtze River Delta and Chengdu-Chongqing urban agglomerations need to curb the overall decline in the absolute levels in both high and low eco-efficiency cities, so policymakers should strengthen the coordination between economic development and ecological environment policy, and promote the integration and optimal allocation of resources within the region. In the meantime, they should also focus on the coordination between urban planning and environmental protection, and firmly implement ecological environment improvement as a long-term goal.

Secondly, reducing intra-regional disparities is the first task in realizing synergetic development in the eco-efficiency of urban agglomeration. Currently, despite the relatively consistent performance of urban agglomeration as a whole in terms of synergy, the bifurcation within urban agglomeration is becoming increasingly obvious. The National New Urbanization Plan (2021–2035) proposes to use urban agglomeration as the main platform to promote coordination and linkage among cross-regional cities in environmental governance. China’s five major urban agglomerations should also seize this opportunity to promote resource sharing and complementary technology transfer by establishing a regional joint prevention and control mechanism for ecological environment governance. To share practical experience and successful cases, strengthen communication and cooperation among cities within the urban agglomeration, and resolve environmental governance issues collaboratively, this mechanism should consist of regular meetings, information sharing, joint actions, etc., such as regular joint meetings and the signing of cooperation agreements. Meanwhile, the incentive mechanism should also be established to make full use of the existing comprehensive network transportation system within the urban agglomeration and to realize the complementary advantages of resources and information integration and interaction, which all lead to the purpose of ensuring long-term and stable implementation of the joint prevention and control mechanism. For example, environmental protection infrastructure, energy resources, technological research, and development results can be shared to reduce the cost of ecological environment governance and to improve overall efficiency. In addition, the government should strengthen the policy guidance and supervision of the cities within the urban agglomeration, strengthen the coordination between policy formulation and implementation, promote technological innovation and exchange, optimize resource allocation within the urban agglomeration, and promote the synergistic improvement of ecological efficiency. At the same time, urban agglomeration should also be equipped with a monitoring and evaluation system for environmental governance to evaluate the results of governance in each city on a regular basis, so that problems can be identified and targeted measures can be taken promptly.

For another, different urban agglomerations should adopt different measures to promote the improvement of eco-efficiency. To promote the level of urban eco-efficiency, different urban agglomerations should adopt different policies and measures. On the whole, all the local governments need to pay more attention to financial input and innovative technology to enhance the level of eco-efficiency, but each urban agglomeration should have its emphasis. The Beijing-Tianjin-Hebei and the middle reaches of the Yangtze River urban agglomeration should pay attention to the optimization and upgrading of the energy structure, and also increase the investment in environmental infrastructure and environmental pollution control within each city. The Yangtze River Delta and Pearl River Delta urban agglomeration should attach importance to the transformation and upgrading of industrial structure to alleviate the spatial imbalance of urban eco-efficiency caused by the imbalance of industrial structure. While increasing the proportion of FDI, the Chengdu-Chongqing urban agglomeration should also focus on improving the fiscal level. In addition, from the perspective of time trends, the importance of population density increased in all the urban agglomerations. The role of population agglomeration should be considered in formulating relevant economic and social development plans to alleviate the spatial imbalance of urban eco-efficiency caused by differences in population density.

## Supporting information

S1 Data (XLS)
